# Tipping the Scales: Quantifying the Impact of USMLE Step 1 Pass/Fail Scoring on Application Interpretation in the Integrated Plastic Surgery Match

**DOI:** 10.1016/j.jsurg.2025.103625

**Published:** 2025-07-21

**Authors:** Cole Bird, Kerilyn N. Godbe, Niaman Nazir, Sterling Braun, Rebecca Farmer, Richard Korentager

**Affiliations:** † University of Kansas School of Medicine, Kansas City, Kansas; § Department of Plastic Surgery, University of Kansas Medical Center, Kansas City, Kansas; ¶ Department of Population Health, University of Kansas Medical Center, Kansas City, Kansas

**Keywords:** step 1, plastic surgery, pass/fail, match, residency, Professionalism, Interpersonal and Communication Skills

## Abstract

**OBJECTIVE:**

The United States Medical Licensing Exam^®^ (USMLE^®^) Step 1 exam implemented pass/fail scoring to “create a more holistic residency application selection process.” We aimed to assess whether this transition favored holistic review or redirected emphasis to other metrics.

**DESIGN:**

Quantifiable data from 330 Plastic Surgery Common Applications (PSCAs) submitted to a single academic center were collected. Applicants were stratified into 2 groups based on numeric or pass/fail Step 1 score. Logistic regression was performed for each group to assess the odds of matching based on quantifiable application components.

**SETTING:**

2023-2024 Match cycle.

**PARTICIPANTS:**

330 integrated plastic surgery applicants.

**RESULTS:**

A total of 137 applications reported numeric Step 1 scores, while 193 reported pass/fail scores. For applicants with a numeric Step 1 score, the factors most predictive of matching were Step 2 score >265 (OR 159 [95% CI, 6.6-999], Step 1 Score between 240 and 250 (OR 21.8 [95% CI, 4.8-98.6]), and being a U.S. citizen (OR 21.1 [95% CI, 3.9-116]). Letter of recommendation numeric strength (OR 11.0 [95% CI, 2.7-45.6]) was also a significant match predictor, followed by more than 16 podium presentations (OR 10.8 [95% CI, 2.6-45.5]). In comparison, for students with pass/fail Step 1 scores, there were only 3 significant factors that predicted a successful match: strength of recommendation letters (OR 3.7 [95% CI, 1.8-7.6]), Alpha Omega Alpha society membership (OR 3.2 [95% CI, 1.3-7.7]), and graduation from a U.S News and World Report top 40 medical school in research (OR 3.0 [95% CI, 1.5-6.1]).

**CONCLUSION:**

For applicants with a numeric Step 1 score, Step 1 and 2 scores remained the most critical match factors. In comparison, letter of recommendation strength and graduation from a top 40 medical school were most important for applicants with pass/fail scoring, indicating a shift toward other application metrics following the scoring change. (J Surg Ed 82:103625. ^©^ 2025 The Authors. Published by Elsevier Inc. on behalf of Association of Program Directors in Surgery.

## INTRODUCTION

The residency selection process for competitive specialties, such as integrated plastic surgery, has historically placed substantial emphasis on standardized test scores, notably the United States Medical Licensing Examination^®^ (USMLE^®^) Step 1.^1^ This emphasis often contributes to considerable stress and anxiety among medical students, and potentially detracts from holistic applicant assessment, leading to excessive focus on exam performance at the expense of broader medical education goals.^2^ As medical education increasingly recognizes the importance of comprehensive evaluation, recent changes in scoring paradigms have aimed to rebalance the assessment of residency applicants.^3–4^

The USMLE^®^ Step 1 transitioned from numeric scoring to a pass/fail scoring system in 2020. According to the American Medical Association, “the goals in switching Step 1 of the United States Medical Licensing Examination^®^ (USMLE^®^) to pass/fail were manifold. Among the chief aims was reducing the burden of exam preparation for medical students and creating a more holistic residency-application selection process.”^5^ Anticipation of this change prompted several studies to explore potential impacts of pass/fail scoring on the residency application process. Survey-based studies of program directors predicted increased emphasis on alternative metrics, such as USMLE^®^ Step 2 scores, research productivity, and strength of letters of recommendation, as programs sought new objective markers to differentiate candidates.^6–7^ However, actual data-driven analyses evaluating these predicted shifts remain sparse, particularly in highly competitive fields.

Our earlier work demonstrated that applicants in the 2023-2024 plastic surgery match who possessed numeric Step 1 scores were more frequently ranked into a high or low tier, while applicants who reported a pass/fail score were placed into the middle tier.^8^ This study demonstrated that differences in application interpretation between numeric and pass/fail Step 1 groups are present, as Step 1 score was the only independent predictor associated with high versus middle tier placement.^8^ However, the study did not investigate whether evaluators placed different emphasis on other key application metrics, such as graduation from a top 40 medical school, research productivity, or honor society status, when considering applicants. We aimed to expand upon this earlier work by objectively evaluating whether the transition to pass/fail Step 1 scoring altered the predictive power of specific application components among integrated plastic surgery applicants. By comparing applicants with numeric versus pass/fail Step 1 scores, we provide essential insights into how residency programs are practically adapting their selection processes, guiding future applicants and program directors toward more informed and equitable application strategies.

## MATERIAL AND METHODS

### Data Collection

Following Institutional Review Board Approval, we conducted a retrospective analysis of 330 Plastic Surgery Common Applications (PSCAs) submitted to the University of Kansas Medical Center during the 2023-2024 residency application cycle. Data extraction was performed as described by Godbe *et al*.^9^ under Applicant Data Collection. Additional data that was collected included number of podium presentations, poster presentations, and plastic surgery standardized letter of recommendation numeric rank (1, 2-5, 5-10, 10-20). Letter numeric strength was derived from the national standardized letter form for integrated plastic surgery residency applicants, in which recommenders ranked applicants against previously ranked applicants from their home institution using defined categories: Number 1, 2-5, 5-10, 10-20 or would not rank. Numerical scores were assigned accordingly (rank 1 = 1 point, 2-5 = 2 points, 5-10 = 3 points, 10-20 =–10 4 points, would not rank = 5 points). Scores from all letters submitted with each application were averaged to determine the applicant’s total letter of recommendation strength.

Each numeric rank was assigned a point value (*i.e*., rank 1 assigned 1 point, rank 2-5 assigned 2 points, rank 5-10 assigned 3 points, rank 10-20 assigned 4 points, and do not rank assigned 5 points). These numeric score assignments for all letters submitted with a single application were then averaged to provide an applicant’s total letter of recommendation strength.

### Statistical Analysis

Categorical variables were summarized using frequencies and percentages, while continuous variables were summarized by medians, means, and quartile ranges. The chi-square test was used to make global comparisons of categorical variables. Student t-tests were utilized to compare statistically significant mean differences between matched and unmatched students. Two-sided *p* values less than 0.05 were considered statistically significant. Categories for continuous variables were determined by comparing the distribution of matched and unmatched applicants to determine the threshold that maximally separated the groups. For example, Step 2 score was split into 4 categories: <235, 235-250, 250-265, and >265. Variable selection for the models included all statistically significant bivariate associations with a *p* value <0.05 and known predictors of matching.

After identification of all predictors with significant differences between matched and unmatched applicants, the 330 applicants were split into 2 groups: those with a numeric Step 1 score, and those with a pass/fail score. Logistic regression models for students with a numeric score and students with a pass/fail score were utilized to determine odds ratios (OR) of matching in each group based on these predictors. The same predictors were used in each model for consistency (with the exception of the addition of numeric Step 1 score for the numeric regression group). The final models were determined by a stepwise elimination; the entrance criterion alpha level was 0.1, and 0.05 was the alpha level needed for a variable to stay in the model. All statistical analyses were conducted using SAS version 9.4 (Copyright (c) 2020 by SAS Institute Inc., Cary, NC, USA. All Rights Reserved).

## RESULTS (NO LIMIT)

Of the 330 applications analyzed, 196 (59.4%) applicants successfully matched. There were no significant differences between the demographics of matched and unmatched applicants other than United States citizenships status (Table 1).^9^ However, significant differences between matched and unmatched applicants were noted regarding AOA status, Step 1 and 2 score, and first and total author publication numbers (Table 2 and 3).^9^ Matched applicants also had a significantly higher number of podium presentation (median 9, IQR 15 vs. median 4, IQR 9; p < 0.0001) and poster presentations (median 8, IQR 7.5 vs. median 5, IQR 8.0; p < 0.0001) than unmatched applicants. Similarly, matched students had an average higher score on standardized letters of recommendation than their unmatched peers (1.9 SD 0.47 vs. 2.4 SD 0.61, p < 0.0001). All continuous variables with significant differences between matched and unmatched students were sorted into categories, with significance persisting in all variables following the new groupings (Table 4).

Among applicants with numeric Step 1 scores, the highest predictors of matching were USMLE^®^ Step 2 scores greater than 265 (OR 159; 95% CI, 6.6-999), Step 1 scores between 240 and 250 (OR 21.8; 95% CI, 4.8-98.6), and U.S. citizenship status (OR 21.1; 95% CI, 3.9-115) (Table 5). Among prior applicants, 73.2% (30/41) had numeric step 1 scores, with a match rate of 29.3% (12/41). Conversely, among new applicants, 36.3% (105/289) reported numeric step 1 scores, with a match rate of 63.7% (184/289). Additional significant factors included Step 2 scores between 251 and 265 (OR 15.3; 95% CI, 1.9-125.9) and 235-250 (OR 15.2; 95% CI, 1.5-151.3), numeric letter of recommendation (LOR) strength (OR 11.0; 95% CI, 2.7-45.5), and podium presentations (more than 16 presentations OR 10.8; 95% CI, 2.6-45.5, 6-10 presentations OR 6.9; 95% CI, 1.3-37.0). In contrast, for applicants with pass/fail Step 1 scores, only 3 significant predictors emerged: strength of recommendation letters (OR 3.7; 95% CI, 1.8-7.6), AOA membership (OR 3.2; 95% CI, 1.3-7.7), and graduation from a top 40 medical school (OR 3.0; 95% CI, 1.5-6.1) (Table 6).

Due to the predictor differences between the numeric and pass/fail Step 1 logistic regression models, an additional comparison of applicant metrics was performed, stratifying based on presence or absence of a numeric Step 1 score (Table 7). There was a significantly higher proportion of re-applicants in the numeric score group (21.9% vs. 5.7%; p < 0.0001) than the pass/fail group. Similarly, more students with numeric scores took a research year (67.2% vs 17.6%; p < 0.0001) and had subsequently higher numbers of podium presentations (p = 0.04) compared to their pass/fail counterparts. A higher proportion of pass/fail applicants, however, graduated from a top 40 medical school (37.3% vs. 26.3%, p = 0.04). There were no other statistically significant differences, including Step 2 score, AOA status, and letter of recommendation strength, between groups. The average Step 1 score of the numeric score group was 241 (SD 15.6, Range 190-270), with matched Step 1 applicants having an average higher score than their unmatched counterparts (246.9, SD 11.0 vs. 233.8, SD 17.3; p < 0.0001).

## DISCUSSION

When the USMLE^®^ transitioned Step 1 scoring from a 3-digit numeric scale to a pass/fail designation, the change immediately sparked debate among medical students, educators, and residency program directors. While the intended aim of this change was to shift emphasis away from standardized test scores and focus on a more holistic approach for evaluating applicants, data within the literature on the actual changes that have occurred are sparse. Our results are the first to quantitatively demonstrate that residency programs, in the absence of numeric Step 1 scores, have significantly altered the factors that they prioritize.

The model presented here for the 2023-2024 numeric Step 1 score cohort yielded similar results to a logistic regression match model by Thomas et al.^10^ which was published in 2023. This group utilized match data from the 2020-2021 application cycle to identify Step 1 score (OR 1.07, CI, 1.04-1.12, p = 0.0002), number of publications (OR 1.21, CI, 1.09-1.39, p = 0.002), and letter of recommendation ranking (OR 0.089, CI, 0.03-0.20, p < 0.0001) as the 3 significant predictors of match.^10^ Notably, this regression excluded Step 2 scores as a metric. Our results reaffirm the persistence of Step 1 scores and strength of recommendation letters as important match metrics. Interestingly, the number of podium presentations replaced the publication number for the 2023-2024 numeric Step 1 applicants. Perhaps this finding reflects the growing emphasis on the research ‘arms race’ in plastic surgery and could indicate that using acceptance for podium presentation as a proxy for quality, is becoming more important for a successful match, as opposed to sheer research publication quantity. It is important to note that at our institution, no specific cutoff for USMLE step scores was utilized, nor was there exclusion based on citizenship status. The high odds ratio observed likely reflects broader national selection trends rather than internal numeric thresholds, emphasizing that our evaluators considered applicants holistically, incorporating multiple metrics beyond standardized scores.

While applicants who reported 3-digit Step 1 scores continued to rely on standardized test performance, namely Step 2 scores, to bolster their applications, those without numeric Step 1 results faced a substantially different evaluation landscape. Residency programs appeared to place greater emphasis on qualitative credentials, such as the strength of letters of recommendation, class rank, honor society memberships (such as Alpha Omega Alpha [AOA]), and graduation from a top 40 medical school. This finding is in alignment with predictions from Lin et al.^11^ and Salari et al.^12^ who hypothesized a substantial shift toward holistic, qualitative evaluations due to concerns that numeric Step 1 scores disproportionally contributed to applicant stress and limited diversity. Lin and colleagues specifically noted that the pass/fail scoring transition might encourage residency programs to adopt broader assessment methods encompassing character, professionalism, interpersonal skills, and overall clinical potential.^11^ Similarly, Salari et al.^12^ anticipated that holistic review methods would gain prominence as residency programs sought richer, more comprehensive evaluations of candidates’ readiness and suitability for advanced medical training. Our findings corroborate these hypotheses, demonstrating that in the absence of Step 1 scores, residency programs shifted their evaluation criteria toward qualitative metrics, specifically highlighting letters of recommendation and institutional prestige. This trend emphasizes the growing importance of holistic applicant characteristics, likely reflecting program directors’ efforts to identify candidates who demonstrate not just academic excellence but also strong clinical judgement and interpersonal competencies.^11,12^

Conversely, initial survey-based studies anticipated that Step 2 scores and research productivity would substantially fill the qualitative assessment gap left by the absence of numeric Step 1 scores.^6,7^ Instead, our data indicates that while Step 2 scores remain important, they do not overshadow the influence of robust letters of recommendation, honor society recognition, and medical school pedigree. This departure from anticipated reliance on Step 2 scores is intriguing and warrants consideration. One possible explanation is that program directors perceive Step 2 scores as limited predictors of residency performance when considered in isolation, especially without the contextual backdrop formerly provided by numeric Step 1 scores. It is also plausible that programs are cautious about over emphasizing another single numeric metric given ongoing discussions about student wellness, exam-related stress, and equity concerns. Furthermore, qualitative credentials, like letters of recommendation and honor society memberships offer richer, multidimensional insights into applicants’ clinical acumen, professionalism, and interpersonal skills. Medical school prestige and ranking comes from a variety of subjective and objective metrics whose value can be debated. As a result, should be taken with a “grain of salt” when being used in holistic reviews. Thus, the transition to pass/fail step 1 scoring appears to have catalyzed a genuine, albeit unexpected, shift toward qualitative, holistic review metrics rather than simply redirecting toward another numeric assessment.

Importantly, these evolving trends have broader implications for both applicants and residency programs. Applicants from schools that are less highly ranked or who lack access to nationally recognized LOR writers may face increased challenges standing out in a pass/fail environment. Letters of recommendation are inherently more subjective than standardized exam scores, with their value often heavily influenced by the author’s reputation and ability to clearly compare applicants to their peers.^4^ Similarly, membership in AOA or other honor societies frequently depends on nuanced, school-specific selection criteria, potentially disadvantaging students at institutions without AOA chapters or with different internal selection practices. These observations highlight the necessity for residency programs to implement more holistic and equitable evaluation strategies. In adapting to a postnumeric Step 1 landscape, program directors should avoid inadvertently disadvantaging qualified candidates who lack traditional pedigree markers. Structured approaches to recommendation letters, including standardized formats and explicit comparative language, could help address these disparities. Transparent communication from programs regarding the relative value of different application components may also assist applicants in effectively tailoring their efforts. To directly address these concerns at our institution, we implemented a structured ‘match committee’ that specifically defined desirable applicant qualities beyond traditional academic metrics. These included teamwork and resilience, each with assigned quantitative points. Applications were independently reviewed and scored by 2 reviewers, with high-scoring candidates advancing for final consideration to be granted interviews.

Just as structured approaches to recommendation letters are important to level the playing field, 1 must also consider a structured approach to the in-person or virtual interview which is the final step in the selection process. If the approach to interviews could be more standardized across interviewers and institutions, candidates could be evaluated more fairly with less conscious and unconscious bias. This approach could also allow for a more robust evaluation of the selection process across multiple institutions.

Finally, although our study provides valuable initial insights, it carries inherent limitations. As a retrospective, single-institution analysis, the generalizability of our findings may be limited. Moreover, applicants who still reported a numeric Step 1 score differed systematically from their pass/fail counterparts: a greater proportion had taken dedicated research years or were re-applicants and therefore took Step 1 before the scoring transition. This timing introduces a natural confounder. Older examinees could have lower Step 1 scores, either because they tested under a more competitive environment, or because individuals with weaker scores are disproportionately likely to extend training to strengthen their applications. Hence, the lower Step 1 scores in our numeric cohort probably reflect this selection effect, rather than a true relaxation of residency programs expectations. Consistent with this interpretation, NRMP data show that mean Step 1 scores for matched plasticsurgery applications fell from 251 (unmatched = 240) in 2021-22 to 247 (unmatched = 226) in 2023-24.^13,14^ These contextual shifts should be considered when evaluating how qualitative metrics influence match outcomes. It is also important to recognize that whether or not a student completed a sub-internship at our institution wasn’t included in the analysis. As sub-internships are well-recognized as playing an important factor in the match process their impact will need to be considered in future analyses especially when looking across multiple programs.

Future analyses directly comparing applicants with numeric scores in the 2022-2023 application cycle to pass/fail applicants in the 2023-2024 cycle in multiple specialties and institutions would help address the inherent bias in the data and strengthen the external validity of our conclusions. Ultimately, broader multi-institutional research projects and ongoing dialogue among stakeholders are essential to ensuring that the transition to pass/fail Step 1 scoring fosters a fair, inclusive, and merit-based residency application environment.

## CONCLUSIONS

The transition to pass/fail Step 1 scoring has clearly reshaped how residency programs evaluate applicants, placing greater emphasis on subjective measures such as the strength of recommendation letters, Alpha Omega Alpha membership, and attendance at highly regarded medical schools. These results stand in contrast to early survey-based predictions of a heavier reliance on Step 2 scores and research productivity, underscoring the need for programs to reevaluate their assessment strategies and ensure that holistic reviews genuinely capture each candidate’s potential. At the same time, medical schools must adapt by prioritizing mentorship, clinical experiences, and other training components that foster the development of strong recommendation letters and well-rounded applicants. Ongoing and broader multi-institutional research will be crucial in refining these insights and helping the medical education community address any unintended biases arising from subjective evaluations, ultimately promoting a more equitable and transparent residency selection process.

## Figures and Tables

**TABLE 1.** Applicant Demographics by Match Status and in Total^9^

Applicant Demographics N (%)	Matched		p Value
	Yes 196 (59.4%)	No 134 (40.6%)	
Gender identity, N (%)			0.10
Male, 147 (44.5)	79	68	
Female, 181 (54.8)	116	65	
Prefer not to identify, 2 (0.6)	1	1	
Race, N (%)			0.11
White, 151 (45.8)	98	53	
Asian, 58 (17.6)	37	21	
Hispanic, 34 (10.3)	17	17	
Black, 24 (7.3)	12	12	
American Indian, 1 (0.3)	0	1	
Other, 14 (4.2)	5	9	
Multiple, 37 (11.2)	23	14	
Prefer not to identify, 11 (3.3)	4	7	
US Citizen, N (%)			**0.02** *
Yes, 281 (85.2)	174	107	
No, 49 (14.8)	22	27	
Multilingual?, N (%)			0.15
Yes, 147 (44.5)	81	66	
No, 183 (55.5)	115	68	
Presence of additional degrees, N (%)			0.23
Masters, 71 (21.5)	38	33	
PhD, 6 (1.8)	4	2	
None, 251 (76.1)	154	97	
Other, 2 (0.6)	0	2	

* Significant at p < 0.05;

** Highly significant at p < 0.01;

*** Very highly significant at p < 0.001.

**TABLE 2.** Comparison of Applicant Honor Society and Board Score Metrics Between Matched and Unmatched Students^9^

Applicant Metrics N(%)	Matched		p Value
	Yes 196 (59.4%)	No 134 (40.6%)	
AOA, N (%)			< 0.0001***
Yes, 86 (26.6)	69 (80.2)	17 (19.8)	
No, 107 (32.4)	54 (50.5)	53 (49.5)	
GHHS, N (%)			0.15
Yes, 42 (12.7)	30 (71.4)	12 (28.6)	
No, 172 (52.1)	102 (59.3)	70 (40.7)	
Step 1 score			< 0.0001***
Mean (standard deviation)	246.9 (11.0)	233.8 (17.3)	
	220–270	190–268	
Range			
Step 2 score			< 0.0001***
Mean (standard deviation)	255.2 (11.7)	246.0 (14.5)	
	223-280	209-274	
Range			

* Significant at p < 0.05;

** Highly significant at p < 0.01;

*** Very highly significant at p < 0.001.

**TABLE 3.** Number of Self-Reported First and Total Author and Total Author Publication, and Poster and Podium Presentations Stratified by Matched and Unmatched^9^

	Match		
	Yes	No	p Value
	Median (IQR)		
First author publications	3 (5)	2 (5)	**0.02** **
Total author publications	8 (11)	5(8)	**0.003** **
Podium presentations	9 (15)	4(9)	< 0.0001***
Poster presentations	8 (7.5)	5(9)	< 0.0001***

Median was utilized for research productivity due to nonparametric distribution of these measures. IQR: interquartile range.

* Significant at p < 0.05;

** Highly significant at p < 0.01;

*** Very highly significant at p < 0.001.

**TABLE 4.** Conversion of Continuous Variables With Statistically Significant Differences Between Matched and Unmatched Applicants Into Categorical Variables (for Inclusion in Logistic Regression Model)

	Match Status		
Applicant Metrics	Yes (59.4%)	No (40.6%)	p Value
Step 1 score, N (%)			< 0.0001***
<240 (N = 37)	16 (21.3)	38 (63.3)	
240-250 (N = 85)	32 (42.7)	10 (16.7)	
251-260 (N = 153)	15 (20.0)	10 (16.7)	
260+ (N = 45)	12 (16)	2 (3.3)	
Step 2 score, N (%)			< 0.0001***
<235 (N = 37)	12 (6.2)	25 (19.7)	
235-250 (N =85)	45 (23.3)	40 (31.5)	
251-265 (N = 153)	98 (50.8)	55 (43.3)	
265+ (N = 45)	38 (19.7)	7 (5.5)	
First author publications, N (%)			**0.01** *
0 (N = 120)	59 (30.1)	61 (45.5)	
1-2 (N = 114)	78 (39.8)	36 (26.9)	
3+ (N = 96)	59 (30.1)	37 (27.6)	
Total author publications, N (%)			**0.01** *
0-3 (N = 153)	80 (40.8)	73 (54.5)	
4-5 (N = 48)	26 (13.3)	22 (45.8)	
6-9 (N = 59)	44 (22.5)	15 (11.2)	
10+ (N = 70)	46 (23.5)	24 (17.9)	
Podium presentations, N (%)			**0.0009** ***
0-5 (N = 146)	71 (36.2)	75 (56.0)	
6-10 (N = 62)	36 (18.4)	26 (19.4)	
11-15 (N = 47)	33 (16.8)	14 (10.4)	
16+ (N = 75)	56 (28.6)	19 (14.2)	
Poster presentations, N (%)			**0.0002** ***
0-3 (N = 79)	35 (17.9)	44 (32.8)	
4-6 (N = 80)	40 (20.4)	40 (29.8)	
7-10 (N = 74)	52 (26.5)	22 (16.4)	
11+ (N = 97)	69 (35.2)	28 (20.9)	
Average LOR score, N (%)			<0.0001***
<2 (N = 121)	94 (48.0)	27 (20.1)	
2+ (N = 209)	102 (52.0)	107 (79.9)	

* Significant at p < 0.05;

** Highly significant at p < 0.01;

*** Very highly significant at p < 0.001.

**TABLE 5.** Significant Predictors for Matching Into an Integrated Plastic Surgery Residency for Students With a Numeric Step 1 Score

Predictor	Odds Ratio [CI]	p Value
New applicant	9.6 [2.4-38.6]	**0.001** **
U.S. citizenship	21.1 [3.9-115.5]	**0.0004** **
Step 1 score 240-250	21.8 [4.8-98.6]	<0.0001***
Step 2 score 235-250	15.2 [1.5-151.3]	**0.02** *
Step 2 score 251-265	15.3 [1.9-125.9]	**0.01** *
Step 2 score >265	159.3 [6.6->999]	**0.002** **
Average LOR score	11.0 [2.7-45.6]	**0.0009** **
Podium presentations 6-10	6.9 [1.3-37.0]	**0.02** *
Podium presentations >16	10.8 [2.6-45.5]	**0.01** *

* Significant at p < 0.05;

** Highly significant at p < 0.01;

*** Very highly significant at p < 0.001.

**TABLE 6.** Significant Predictors for Matching Into an Integrated Plastic Surgery Residency for Students With a Pass/Fail Step 1 Score

Predictor	Odds Ratio [CI]	p Value
Graduate from top 40 medical school	3.0 [1.5-6.1]	**0.002** **
Inducted into AOA	3.2 [1.3-7.7]	**0.01** *
Average LOR score	3.7 [1.8-7.6]	**0.0005** ***

* Significant at p < 0.05;

** Highly significant at p < 0.01;

*** Very highly significant at p < 0.001.

**TABLE 7.** Comparison of Quantifiable Application Metrics Between Applicants With a Numeric and a Pass/Fail Step 1 Score

Applicant Metrics	Step 1 Score Type		p Value
	Numeric Score (58.7%)	Pass/Fail Score (41.3%)	
Application status, N (%)			< 0.0001***
First time applicant (N = 289)	107 (78.1)	182 (94.3)	
Re-applicant (N = 41)	30 (21.9)	11 (5.7)	
US citizenship, N (%)			0.25
Yes (N = 281)	113 (82.5)	168 (87.0)	
No(N = 49)	24 (17.5)	25 (13.0)	
Medical school rank, N (%)			**0.04** *
< 40 (N = 108)	36 (26.3)	72 (37.3)	
≥40 or unranked (N = 222)	101 (73.7)	121 (62.7)	
Presence of home program, N (%)			**0.08** *
Yes (N = 166)	61 (44.5)	105 (54.4)	
No(N = 164)	76 (55.5)	88 (45.6)	
AOA, N (%)			0.15
Yes (N = 86)	29 (21.1)	57 (29.5)	
No(N = 107)	51 (37.2)	56 (29.0)	
Completed a research year, N (%)			< 0.0001***
Yes (N = 126)	92 (67.2)	34 (17.6)	
No (N = 204)	45 (32.8)	159 (82.4)	
Step 1 score			-
<240	54 (40.0)	-	
240-250	42 (31.1)	-	
251-260	25 (18.5)	-	
>260	14 (10.4)	-	
Step 2 score, N (%)			0.85
<235 (N = 37)	14 (10.5)	23 (12.3)	
235-250 (N = 85)	34 (25.6)	51 (27.3)	
251-265 (N = 153)	64 (48.1)	89 (47.6)	
265+ (N = 45)	21 (15.8)	24 (12.8)	
First author publications, N (%)			0.32
0 (N = 153)	47 (34.3)	73 (37.8)	
1-2 (N = 48)	44 (32.1)	70 (36.3)	
3+ (N = 59)	46 (33.6)	50 (25.9)	
Total author publications, N (%)			0.60
0-3 (N = 153)	60 (43.8)	93 (48.2)	
4-5 (N = 48)	19 (13.9)	29 (15.0)	
6-9 (N = 59)	24 (17.5)	35 (18.1)	
10+ (N = 70)	34 (24.8)	36 (18.7)	
Podium presentations, N (%)			**0.04** *
0-5 (N = 146)	53 (38.7)	93 (48.2)	
6-10 (N = 62)	21 (15.3)	41 (21.2)	
11-15 (N = 47)	23 (16.8)	24 (12.4)	
16+ (N = 75)	40 (29.2)	35 (18.1)	
Poster presentations, N (%)			0.48
0-3 (N = 79)	33 (24.1)	46 (23.8)	
4-6 (N = 80)	29 (21.2)	51 (26.4)	
7-10 (N = 74)	29 (21.2)	45 (23.2)	
11+ (N = 97)	46 (33.6)	51 (26.4)	
Average LOR			0.86
<2 (N = 121)	51 (37.2)	70 (36.3)	
2+ (N = 209)	86 (62.8)	123 (63.7)	
Match status, N (%)			0.15
Yes (N = 196)	75 (54.7)	121 (62.7)	
No(N = 134)	62 (45.3)	72 (37.3)	

* Significant at p < 0.05;

** Highly significant at p < 0.01;

*** Very highly significant at p < 0.001.
